# Investigate the Binding of Catechins to Trypsin Using Docking and Molecular Dynamics Simulation

**DOI:** 10.1371/journal.pone.0125848

**Published:** 2015-05-04

**Authors:** Fengchao Cui, Kecheng Yang, Yunqi Li

**Affiliations:** Key Laboratory of Synthetic Rubber & Laboratory of Advanced Power Sources, Changchun Institute of Applied Chemistry (CIAC), Chinese Academy of Sciences, Changchun, P. R. China; Russian Academy of Sciences, Institute for Biological Instrumentation, RUSSIAN FEDERATION

## Abstract

To explore the inhibitory mechanism of catechins for digestive enzymes, we investigated the binding mode of catechins to a typical digestive enzyme-trypsin and analyzed the structure-activity relationship of catechins, using an integration of molecular docking, molecular dynamics simulation and binding free energy calculation. We found that catechins with different structures bound to a conservative pocket S1 of trypsin, which is comprised of residues 189–195, 214–220 and 225–228. In the trypsin-catechin complexes, Asp189 by forming strong hydrogen bonding, and Gln192, Trp215 and Gly216 through hydrophobic interactions, all significantly contribute to the binding of catechins. The number and the position of hydroxyl and aromatic groups, the structure of stereoisomers, and the orientation of catechins in the binding pocket S1 of trypsin all affect the binding affinity. The binding affinity is in the order of Epigallocatechin gallate (EGCG) > Epicatechin gallate (ECG) > Epicatechin (EC) > Epigallocatechin (EGC), and 2R-3R EGCG shows the strongest binding affinity out of other stereoisomers. Meanwhile, the synergic conformational changes of residues and catechins were also analyzed. These findings will be helpful in understanding the knowledge of interactions between catechins and trypsin and referable for the design of novel polyphenol based functional food and nutriceutical formulas.

## Introduction

**Fig 1:**
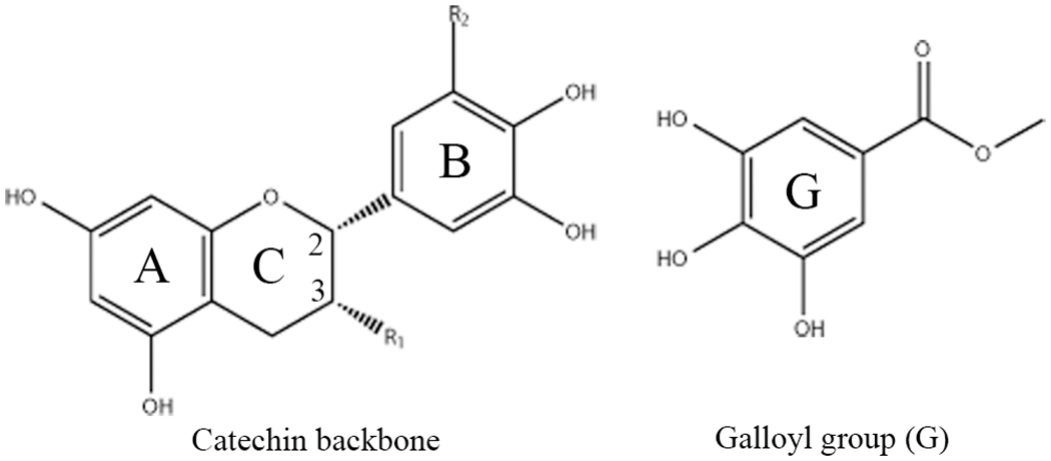
Molecular structures of catechins. Molecular structures of catechins in the tea polyphenol. EGCG: R2 is OH, R1 is G; ECG: R2 is H, R1 is G; EGC: R1 and R2 are OH; EC: R1 is OH, R2 is H.

Catechin, a major component of tea polyphenol, has shown various benefits in health promotion. It has four subtypes including (-)-epigallocatechin gallate (EGCG), (-)-epigallocatechin (EGC), (-)-epicatechin gallate (ECG), and (-)-epicatechin (EC) (See Fig 1 for details). Epidemiological and clinical studies have indicated that catechins have positive contributions in reducing the risk of several kinds of cancers, such as lung cancer[1,2], gastrointestinal cancer[3,4], skin cancer[5], and liver cancer[6] etc. Formulation of catechins to inhibit cardiovascular diseases[7–9] and fight against diabetes and obesity[10,11] also attracted a number of attentions. It is believed that the strong binding affinity with many functional proteins and the free radical scavenge capability of catechins are the source of its bioactivities. The strong binding affinity of catechins to proteins can prohibit beta-Amyloid, thus suppress the associated disease such as Parkinson's disease (PD), Alzheimer's disease (AD) etc[12–14]. It also can provide up to five times stronger free radical scavenge capability than that of vitamin C or E[15]. Comparing to intensive studies on the bioactivities of catechins, clear presentation of the molecular mechanism regarding to catechins binding to functional proteins is still absent.

Generally, catechins and proteins have strong interactions through hydrogen bonding and/or hydrophobic attractions. They can form soluble complexes which further aggregate and eventually lead to precipitation[16]. At molecular scale, the strong binding of catechins may alter the natural conformation of proteins or block the interaction of the natural substrate with proteins, and thus perturb the stability and bioactivity of proteins. For example, the bioactivities of trypsin[15,17,18], α-chymotrypsin[18], decarboxylase[19], squalene epoxidase[20], ribonuclease[21], α-amylase[15,22], xanthin oxidase[23], histidine/dopa decarboxylase[24] and human salivary α-amylase[25] can be effectively suppressed in the presence of catechins. In these proteins, trypsin, which is a typical globular digestive enzyme responsible for hydrolyzing proteins, is expressed in various tissues and cancer cells[26,27]. Activated trypsin plays a pivotal role in digesting food molecules to provide enough nutrients for human body, while the excessive activity of trypsin is involved in the proliferation, invasion, and metastasis of tumor cells through degrading the extracellular matrix[28,29]. A balanced activity of trypsin is very necessary for express its different physiological functions. The major bioactivity of trypsin, i.e., the hydrolysis of the peptide backbone at the carboxyl side of lysine or arginine, is charged by a collaboration of the catalytic triad (His57, Asp102 and Ser195) in the active site, which is a preserved feature in the family of serine proteases[30,31]. This active site, also named as the S1 pocket, is composed by residues 189–195, 214–220 and 225–228. It has been reported that tea polyphenol mainly comprising of catechins can inhibit the digestive activity of trypsin[15,17]. The strong interaction between catechins and trypsin can significantly change the secondary structure and the size of trypsin[18,32]. However, molecular mechanism regarding to the interaction in the binding pocket and the change in protein conformation related to the bioactivity suppression is still far from clear. Meanwhile, the interaction of EGCG with different functional proteins has been widely reported, but researches on catechins with different chemical structures and stereoisomers are limited. Therefore, the clarification of the structure-activity relationship of catechins and its binding behavior to trypsin can help to understand the regulation of the hydrolyzed activity of trypsin through the introduction of catechins into food or drug, which is critical to minimize antinutritional effects and reduce cancer risks.

In the present study, molecular docking, molecular dynamics simulation and binding free energy calculations have been performed to explore the binding mechanism of catechins to trypsin. These computational approaches have impressively help the structure-based drug design and the molecular mechanism exploration associated with bioactivities through the consistency in binding sites and binding affinities over a number of protein complexes[33–36]. Semi-flexible docking was used to determine binding sites, followed by fully flexible atomic molecular dynamics simulation to refine the complex structures. The refined complex structures were then engaged for binding free energy calculation using molecular mechanics-Poisson Boltzmann surface area (MM-PBSA) method. Decomposition of binding free energies was also preformed for fundamental understanding the binding of catechins to trypsin at the residue level. Finally, impacts from different structures and stereoisomers were discussed and conclusion was drawn.

## Computational Details

### Initial Structure Construction

Initial structure of trypsin was taken from Protein Data Bank (PDB)[37] (www.rcsb.org), i.e., 2PTN with 1.55 Å resolution[38]. Water molecules in the structure were removed and hydrogen atoms were added using Schrӧdinger[39]. The protonation states of residues (His, Asp, Glu, Arg, Lys, N- and C-terminus) were set at pH 7.0 according to experimental determinations[40,41]. All residues of protein were then parameterized using the AMBER03 force field[42]. The structures of EGC and EGCG were retrieved from PDB, and the structures of EC and ECG were obtained by replacing the corresponding hydroxyl group from EGC and EGCG with hydrogen atom, respectively. The stereoisomers of EGCG (shown in S1 Fig) were constructed using GaussView5 (http://www.gaussian.com/g_prod/gv5.htm). All these catechin structures then were optimized using B3LYP[43–48] with 6-31G(d,p) basis set implanted in Gaussin09[49]. The partial charges of atoms in catechins were determined by R.E.D. (RESP ESP charge Derive)[50], and other atomic parameters were assigned according to general AMBER force field (GAFF)[51].

### Molecular Docking

Molecular docking of each catechin to trypsin was carried out by AutoDock Vina[52], in which the Iterated Local Search Globule Optimizer[53,54] was applied to locate the most favorable binding site. Semi-flexible docking method was used, where trypsin was treated as a rigid body and all rotatable bonds in the catechins were sampled. Optimal binding sites were searched in a box of 60×60×60 Å^3^ that covered the entire exterior of the protein. The box had 1.0 Å grid spacing and centered at the geometric center of the protein. In each docking experiment, top 20 models were selected according to the binding affinity calculated by the scoring function in AutoDock Vina.

### Molecular Dynamics Simulation

Trypsin-catechin complex structures from docking were further refined in a fully flexible atomic molecular dynamics simulation using NAMD (version 2.9)[55]. A cubic TIP3P water box was used to enclose structure models with a ~10 Å buffering in all three orthogonal dimensions. As a result, the water box size of all systems is ca. 67×62×72 Å, while the total number of atoms are approximately 27000 atoms. We also added 8 Cl^-^ ions and 14 pairs Na^+^/Cl^-^ ions to hold charge neutralization and 0.1 M monovalent ionic strength, respectively. In order to exhaustively sample complex structures while keep protein structures close to the native conformation, six phases as listed in Table 1 were carried out. All three phases of minimization run 5000 steps conjugate gradient energy minimization, including solvent relaxation with both protein and ligand structures fixed, ligand and protein side chain relaxation with protein backbone fixed, and relaxation of all atoms. Then the system was gradually heated to 310 K in a 220 ps relaxation. Subsequently, NPT ensemble equilibration was carried out to achieve the initial configuration equilibrium before a 30 ns NVT dynamics simulation to generate the trajectories of complex structures for analysis. Here it is worthy to note that we have run an 80 ns MD simulation on the complex. We found that the structural fluctuations of both protein and ligand revealed from the RMSD are quite small (see S2 Fig) and the binding state has no change during the simulation time, so we keep on 30 ns simulation through all this work. In each simulation trajectory, 2000 complex structures from the last 20 ns MD simulation at a time interval of 10 ps were extracted and used for complex structure analysis and binding free energy calculation. Further, we clustered these structures using SPICKER[56] based on the RMSD value of backbone atoms, and the centroid structure of the largest cluster was selected as the typical model for illustrating complex structure.

**Table 1:** Molecular dynamics simulation settings.

Phase	Steps/Time	Temp(K)	Fixed (Y or N)				Ensemble
			Backbone	Side-chains	Ligand	Solvent	
Minimization	5000	0	Y	Y	Y	N	NVT
Minimization	5000	0	Y	N	Y	N	NVT
Minimization	5000	0	N	N	N	N	NVT
Heat	220 ps	0–310 K	N	N	N	N	NVT
Equilibration	1 ns	310	N	N	N	N	NPT
Production	30 ns	310	N	N	N	N	NVT

Langevin thermostat was used to maintain the temperature at 310 K with the dampening coefficient of 5 ps^-^. Pressure was scaled at 1 atm with Nosé-Hoover Langevin piston method [57–60] with the piston period of 100 fs, the piston decay of 50 fs, and the piston temperature at 310 K. Periodic boundary conditions were applied and long-range electrostatics were treated using the particle-mesh Ewald (PME) method[61,62]. Non-bonded interactions were calculated using a cutoff of 12 Å without switch function, and a 14 Å neighbor list was updated every 10 steps of the dynamics. All covalent bonds involving hydrogen atoms were confined by SHAKE algorithm[63,64]. The integrated time step is set to 2 fs, and the coordinates of trajectories were saved every 1 ps throughout all MD simulations.

The convergence of MD simulations on the trypsin-catechins complexes was evaluated by the root mean-square deviation (*RMSD*) of atoms after superposition, which is defined by

$$RMSD=\sqrt{\frac{1}{N}\sum_{i=1}^{N}{\left(r_{i}-r_{i,ref}\right)}^{2}}$$ (1)

where *N* is the number of atoms, *r*_*i*_ and *r*_*i*,*ref*_ is the position of atom *i* in a structure and in a referent structure, respectively. In the analysis of simulation trajectories, the PDB structure of trypsin and the best docking structure of catechins were selected as the referent structure. The conformation change and structure fluctuation of protein during simulations also can be traced by the radius of gyration (*R*_g_), the solvent accessible surface area (SASA) and the B-factor. The *R*_*g*_ was calculated by

$$R_{\text{g}}=\sqrt{\sum_{i=1}^{N}m_{i}{\left(r_{i}-\bar{r}\right)}^{2}/\sum_{i=1}^{N}m_{i}}$$ (2)

where *m*_*i*_ and *r*_*i*_ are the mass and the position of the *i*th atom, respectively, and *$\bar{r}$* is the mass center. The SASA was computed by the maximal speed molecular surfaces algorithm[65] implemented in visual molecular dynamics (VMD[66]), using a probe radius of 1.4 Å. The B-factor was defined as

$$B_{k}=\frac{8}{3}\pi^{2}{\left(RMSF_{k}\right)}^{2}$$ (3)

here, *RMSF*_*k*_ is the root-mean-square fluctuation of the atom *k*. It can be calculated through Eq (1), in which *N* is the number of trajectory and *r*_*i*,*ref*_ is the average position of the atom *k* over all trajectories generated in a MD simulation.

### Binding Free Energy Calculation

We extracted the 200 models evenly from the last 20 ns MD trajectories to compute the binding free energy between trypsin and catechins, using the molecular mechanics Poisson-Boltzmann solvent accessible surface area (MM-PBSA) method[67]. The total binding free energy can be calculated by

$$\begin{array}{l} \Delta G_{bind}=\Delta E_{MM}+\Delta G_{sol}-T\Delta S\\ \;=(\Delta E_{vdW}+\Delta E_{ele}+\Delta E_{\mathrm{int}})+(\Delta G_{polar}+\Delta G_{nonpolar})-T\Delta S \end{array}$$ (4)

here, *ΔE*_*MM*_ is the change of molecular mechanical energy, *ΔG*_*sol*_ is the solvation free energy and *TΔS* considers the penalty of entropy. Considering that most protein-polyphenol complexes are enthalpy dominant[68] and the low reliability and high computationally cost[69,70] in entropy computation, we ignored the entropy contributions in the present work. The *ΔE*_*MM*_ includes non-bonded interaction, van der Waals *ΔE*_*vdW*_ and electrostatic *ΔE*_*ele*_ interactions, and local bonded interaction *ΔE*_*int*_. The last term, which is a sum of bond, angle, and dihedral contributions, is counteracted in single trajectory approach. The solvation free energy *ΔG*_*sol*_ contains electrostatic/polar (*ΔG*_*polar*_) and nonpolar (*ΔG*_*nonpolar*_) terms. The nonpolar term can be further divided into the excluded volume contributed by repulsive interaction (*ΔG*_*enpolar*_) and the attractive interaction (*ΔG*_*edisper*_) aroused from the solute-solvent van der Waals dispersion interaction.

The *ΔE*_*MM*_ and *ΔG*_*sol*_ were calculated using Amber[71] and the PBSA module in AmberTools[72], respectively. In the MM-PBSA calculation, a grid spacing of 0.5 Å was employed and the relative dielectric constant was set to 80.0 at the exterior and 2.0 at the interior of catechin-trypsin complex. At last, free energy decomposition at residue level (*ΔG*_*per-decomp*_) also was carried out to provide detailed information of binding site and binding affinity.

## Results and Discussion

### Docking of catechin and trypsin

Based on the 20 top ranked docking models for each complex, the strongest binding affinities and the occurrence of catechins in the S1 pocket with given orientations were computed and summarized in Table 2. Typical models with the strongest binding affinities of different catechins were shown in S3 Fig. It can be seen that the majority of binding of catechins occurred at S1 pocket, ranging from 100.0% for EGCG to 52% for EC. Different functional groups (ring A+C, ring B or ring G) of catechins also exhibit different proneness to closely contact with S1 pocket. Such proneness is in the order of ring G > ring B > ring A+C for EGCG, while the occurrence of ECG in the S1 pocket is ring G > ring A + C > ring B. For EC and EGC, ring B presents in the S1 pocket with higher probability, in agreement with our recent finding in Monte Carlo simulation of catechin-serum albumin complex[73]. By comparing catechins with and without the galloyl group, it can be found that the galloyl group can significantly enhance the binding affinity. This is consistent with that catechins with the galloyl group show much stronger binding and inhibitory ability to various functional proteins (such as human serum albumin and dopa decarboxylase)[19,74,75]. Overall, the binding affinity of catechins to trypsin is in the order of EGCG ≈ ECG > EC ≈ EGC.

**Table 2:** The binding affinity from semi-flexible docking (kcal/mol) and the possibility (in parenthesis) of four types of catechins and their chemical groups binding to the S1 pocket of trypsin.

	EC (52.6%^a^)	EGC (75.0%^a^)	ECG (90.0%^a^)	EGCG (100.0%^a^)
Ring G	—	—	-8.4(61.1%^b^)	-8.5(45.0%^b^)
Ring B	-7.5(70.0%^b^)	-7.3(73.3%^b^)	-8.1(16.7%^b^)	-8.3(40.0%^b^)
Ring AC	-7.0(30.0%^b^)	-6.5(26.7%^b^)	-7.9(22.2%^b^)	-7.5(15.0%^b^)

^a^ The possibility of ligand binding to the S1 pocket.

^b^ The possibility of different groups in each ligand binding to the S1 pocket.

According to the structure models shown in S3 Fig, residues in or at the vicinity of binding site, i.e., the S1 pocket in trypsin, stabilize catechins through hydrogen bond or hydrophobic interactions. With all the four catechins, residues Ser190, Gln192, Ser195 and Val213-Ser214-Trp215 always contribute conserved hydrogen-bond interactions or hydrophobic contacts. Besides, Phe41, Cys42 and Leu99 are involved in hydrophobic interactions, and the backbone of Phe41 forms hydrogen-bond interaction with ECG and EGCG. Catechins, which occupy the catalytic pocket S1 with strong interaction with the residues, hinder the natural substrate binding to trypsin. This indirectly implies that catechins can suppress the hydrolyzed activity of trypsin, in line with experimental observation[15,17,18].

Further, since the 2, 3 carbon atoms in catechins are chiral, each catechin has four stereoisomers, i.e., 2R-3R, 2R-3S, 2S-3R and 2S-3S. The binding affinity and occurrence in the S1 pocket with given orientations for the stereoisomers of EGCG binding to trypsin were summarized in S1 Table, and the docking structure with the superposition of stereoisomers was illustrated in S4 Fig. The natural configuration in tea, 2R-3R EGCG, shows the strongest binding affinity to trypsin, and the S1 pocket is still a conservative binding site for different stereoisomers. For all stereoisomers, rings G and B always show better preference than ring A+C to closely contact with the binding site.

### Molecular dynamics simulation

**Fig 2:**
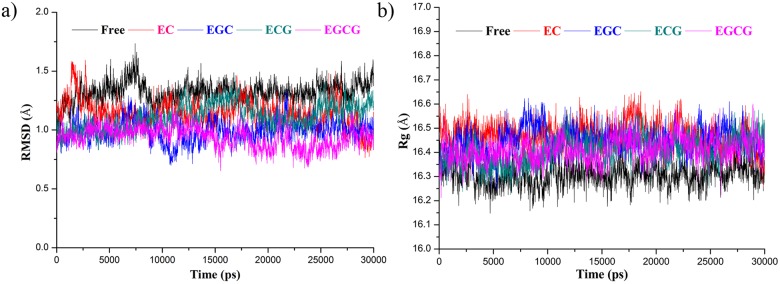
Estimation of MD simulation equilibration and analysis of the stability of protein structure. Time evolutions of a) the backbone RMSD and b) the radius of gyration (*R*_*g*_) of trypsin in MD simulations. Black color indicates trypsin in catechin-free form; red, blue, dark-cyan and magenta indicate trypsin in the complex with EC, ECG, EGC and EGCG, respectively.

The 30 ns MD simulations initialized from representative trypsin-catechin complex structures from docking and the catechin-free trypsin structure were carried out. Time evolutions of RMSD and *R*_g_ of trypsin were displayed in Fig 2. The RMSD vs. simulation time is an important profile to estimate the equilibration procedure in the simulation trajectory and the stability of protein structure upon the binding of ligand[76–78]. Our results indicate that the catechin-free and the catechin-complex systems achieve equilibrium in 10 ns simulation. Therefore, we evenly selected 2000 structure models from the last 20 ns to analyze the binding mode of catechins to trypsin. The average fluctuations represented by the RMSD for the catechin free-trypsin, EC-trypsin, EGC-trypsin, ECG-trypsin and EGCG-trypsin are 1.32 ± 0.09, 1.15 ± 0.11, 1.00 ± 0.09, 1.11 ± 0.11, and 0.94 ± 0.09 Å, respectively. The decrease of RMSD in the complex comparing to the catechin-free trypsin suggests that the stability of the trypsin structure is enhanced upon the binding of catechins. While the fact that the *R*_g_ values in the complex are always larger than the catechin-free trypsin suggests that the enhancement in the stability is not a result of protein becoming more compact, but a synergic conformation change to closely contact with catechins. The average solvent accessible surface area (SASA) of trypsin in the form of free-trypsin, EC-trypsin, EGC-trypsin, ECG-trypsin and EGCG-trypsin were 9702, 9921, 9927, 9813 and 9832 Å^2^, respectively. It further confirmed that protein have conformational changes at given regions to facilitate the binding with catechins, and fully flexible MD simulation is indispensible to reveal ligand binding to protein over semi-flexible molecular docking.

**Fig 3:**
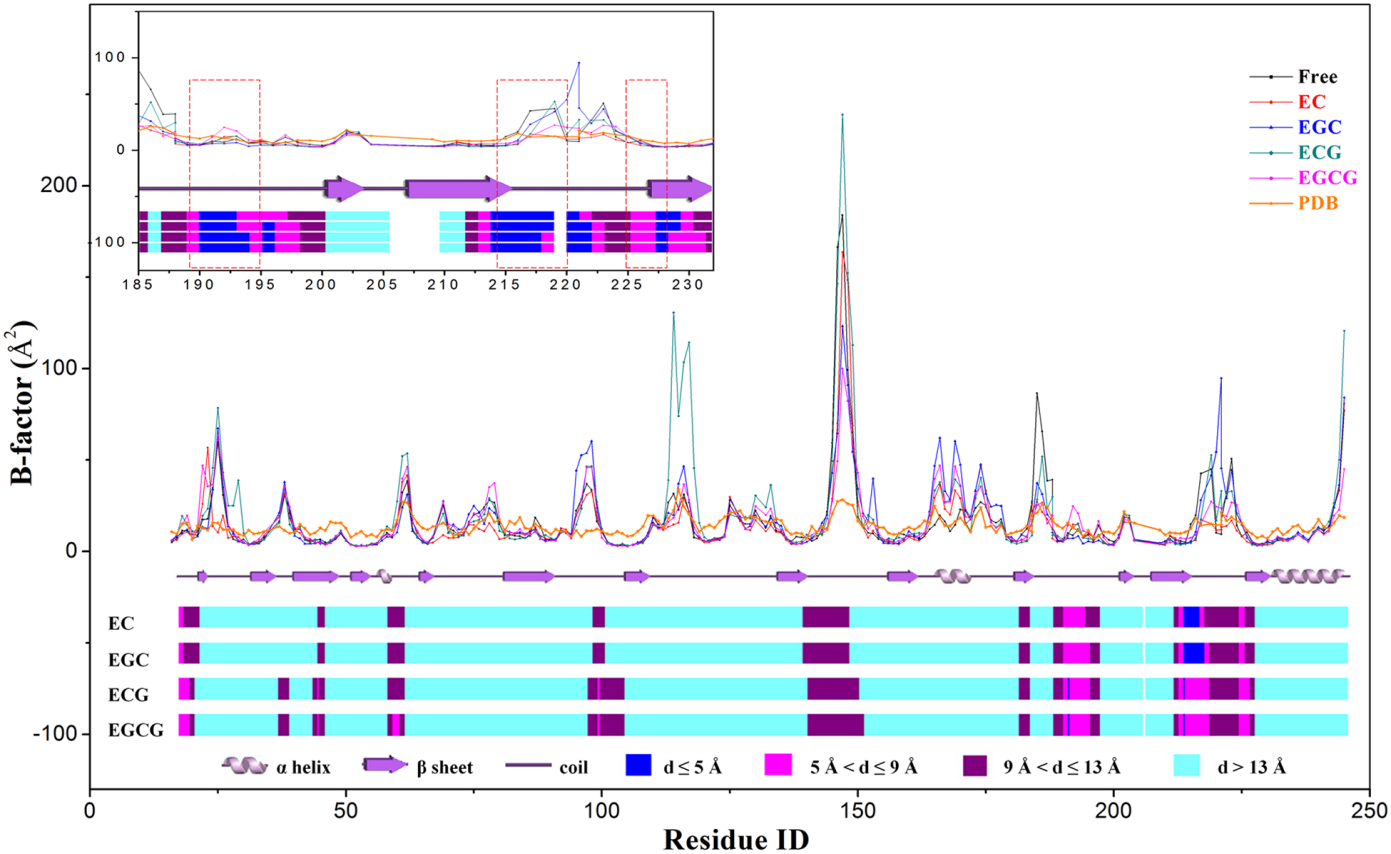
Characterization of residues flexibility. The Cα B-factor for each residue in trypsin computed from MD simulation trajectories in the form of catechin-free (black) and complex with EC (red), ECG (blue), EGC (dark-cyan) and EGCG (magenta), respectively. The orange line represents the Cα B-factor from PDB file. The wiring diagram shows the secondary structure of trypsin. The bar chart at the bottom of picture shows the distance range of the Cα atom to the nearest heavy atom of catechins. The inset enlarges the sequence motifs in the S1 pocket.

To find out the flexibility of residues upon the binding of catechins, Cα B-factor for each residue in trypsin was computed and presented in Fig 3. It can be observed from Fig 3 that the B-factor profile of catechin-free trypsin is similar as the one from X-ray crystallographic measurement saved in the PDB file. This confirms the reliability to assess flexibility of residues using MD simulation. Further, residues within coil always show higher B-factor value, in agreement with that the residues located at the coil are more flexible. Although binding of catechins only leads to slightly change the whole B-factor profile, B-factors of residues 24–27, 37–39 and 96–99 have a remarkable increase. None of these regions is in, but at the vicinity of the S1 pocket. Instead, the majority of residues with close contact with catechins have low B-factor value. It clearly demonstrates that trypsin has a local synergic conformation change upon the binding of catechins. The most protruding region is the residues from 145 to 150 in trypsin with high B-factor in any forms. The first motif of residues in the S1 pocket (residues 189–195) with a low B-factor is similar both in the complex and catechin-free trypsin. The other motif in S1 pocket of residues 214–220 has high B-factor values, and show fingerprints for catechins with different structures.

**Fig 4:**
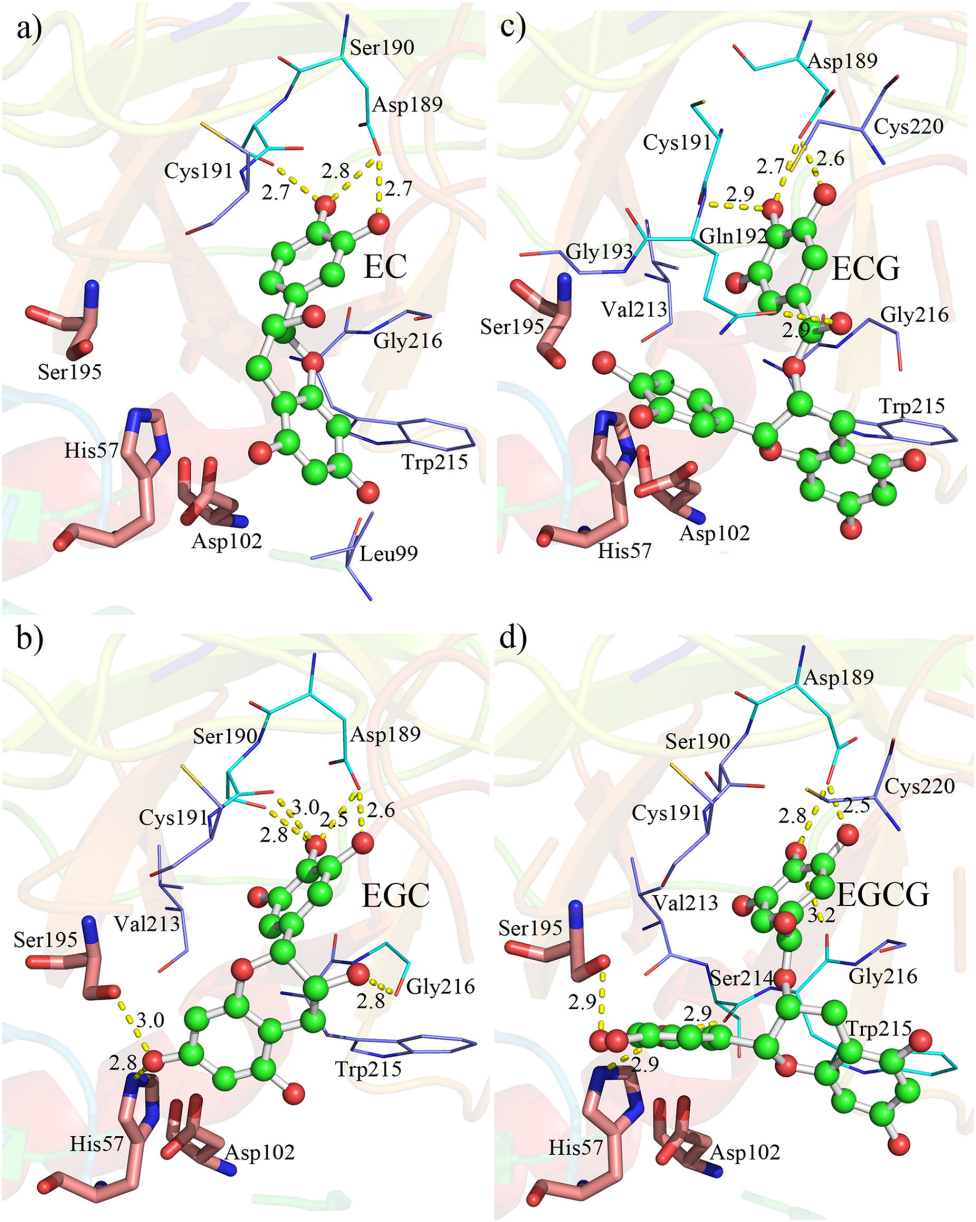
Representative trypsin-catechin complex structures. Representative structure models clustered from MD simulation trajectories for trypsin complex with a) EC, b) EGC, c) ECG and d) EGCG. Catechins are shown as ball-and-stick model, trypsin as cartoon. The catalytic triad (Asp102, His57, Ser195) is shown in stick. Residues interact with catechins by hydrogen bond and hydrophobic interaction highlighted by lines.

**Fig 5:**
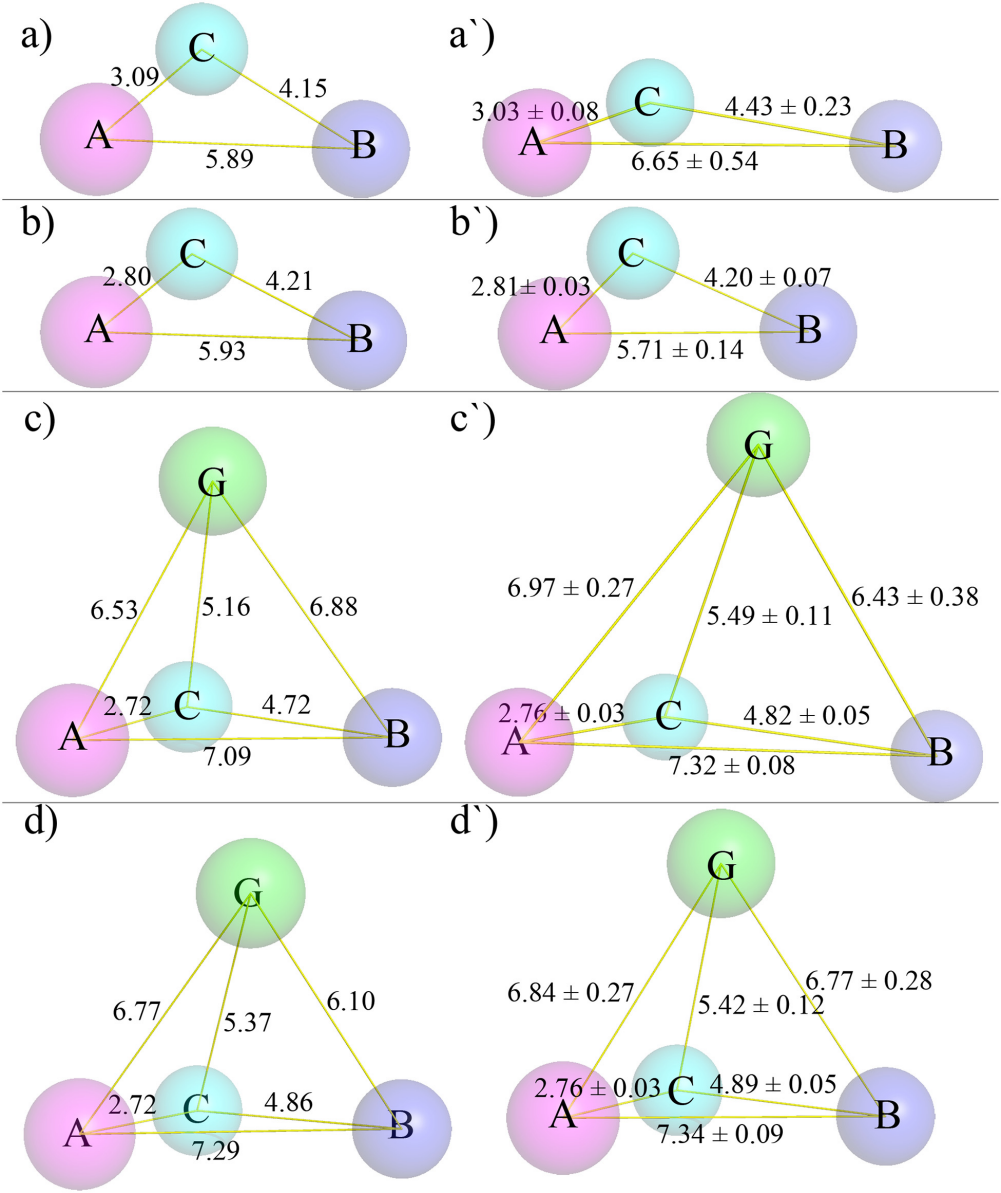
Characterization of the conformation changes of catechins. The distances among the rings of catechins in the optimized structure (a, b, c and d) and their average distances calculated from MD trajectories (a′, b′, c′ and d′). (a and a′) EC; (b and b′) EGC; (c and c′) ECG; (d and d′) EGCG.

The most favorable complex structures were shown in Fig 4. Consistent with the docking results, residues Asp189 and/or Ser190 conservatively form strong hydrogen bond with ring B in EC and EGC, but ring G in ECG and EGCG. Accordingly, the catalytic triad (His57, Asp102 and Ser195) gets close contact with ring A+C in EC and EGC, while ring B in ECG and EGCG. This difference in the structure may result in stronger binding affinity of ECG and EGCG than that of EC and EGC without galloyl groups, in consistent with the results from docking. Comparing with docking structure, other noticeable difference is that residues 41 and 42 no longer show strong interaction with ring B in catechins. Meanwhile, catechins also have synergic conformation changes to accommodate the binding pocket of trypsin. The change of conformation can be viewed from the changes of distances among the rings in catechins, in comparison with their native structure as shown in Fig 5. The change of distance between ring A and ring B is significant with the change of 0.76Å, -0.22Å, 0.23Å and 0.05Å for EC, EGC, ECG and EGCG after binding with trypsin, respectively. Ring G is always stretched to form strong hydrogen bond with the residues in the binding pocket. These results clearly demonstrate that both protein and ligand have remarkable conformation changes to facilitate the binding, and fully flexible simulation is necessary to explore the detailed complex structure. In addition, time evolutions of RMSD of catechins are also plotted in the S5 Fig. It can be seen that the average fluctuations of EC, ECG and EGCG are larger than that of trypsin, while lower for EGC. This indicates that EC, ECG and EGCG are more flexible than trypsin in the complex, while it is trypsin in the trypsin-EGC complex.

### Binding free energy calculation

Based on the MD simulation trajectories, binding free energies of catechins to trypsin were calculated using MM-PBSA method. Since the enthalpy dominates the binding of polyphenol to protein in most instances[68] and low reliability and high cost in entropy computation, the contributions of entropy in the binding free energy were neglected in this investigation. The free energy components were decomposed. The binding free energy and the contributions of its components for each complex were summarized in Table 3. The lowest binding free energies of EC, EGC, ECG and EGCG to trypsin were -12.5, -12.0, -12.8, and -13.1 kcal/mol, respectively. EGCG and ECG have stronger binding affinity than EC and EGC, which is consistent with previous docking results. The binding free energy of catechins to trypsin with different orientations is in the order of ring G > ring B > ring A+C, which agrees with the proneness of them in close contact with the binding pocket. The native stereoisomer of EGCG (2R-3R EGCG) also shows the lowest binding free energy.

**Table 3:** Binding free energies (kcal/mol) and the energy components for catechins with different structures, orientations and stereoisomers.

complexes		ΔE_vdw_	ΔE_ele_	ΔG_polar_	ΔG_enpolar_	ΔG_edisper_	ΔG_bind_
Ligands	Orientation						
EC	Ring B	-27.6(0.2)	-25.9(0.2)	24.5(0.2)	-19.3(0.1)	35.8(0.1)	-12.5(0.2)
	Ring A+C	-32.1(0.2)	-9.3(0.3)	19.7(0.2)	-20.1(0.1)	36.3(0.1)	-5.5(0.2)
EGC	Ring B	-29.6(0.2)	-32.0(0.3)	32.5(0.2)	-21.9(0.1)	39.0(0.1)	-12.0 (0.3)
	Ring A+C	-29.1(0.3)	-13.8(0.2)	18.2(0.1)	-18.3(0.1)	33.0(0.1)	-10.0(0.2)
ECG	Ring G	-40.3(0.3)	-19.3(0.3)	25.4(0.2)	-26.8(0.1)	48.2(0.1)	-12.8(0.3)
	Ring B	-32.6(0.3)	-28.9(0.3)	29.8(0.2)	-23.4(0.1)	44.4(0.1)	-10.7(0.3)
	Ring A+C	-32.5(0.2)	-6.4(0.2)	19.0(0.3)	-19.2(0.1)	39.0(0.2)	-0.1(0.3)
EGCG(2R,3R)	Ring G	-41.1(0.2)	-28.4(0.4)	32.8(0.2)	-27.4(0.1)	51.0(0.1)	-13.1(0.3)
	Ring B	-32.0(0.4)	-7.6(0.3)	16.5(0.2)	-20.3(0.2)	37.3(0.3)	-6.1(0.2)
	Ring A+C	-43.8(0.4)	-9.2(0.2)	21.9(0.2)	-25.6(0.1)	48.9(0.2)	-7.8(0.3)
EGCG(2R,3S)	Ring G	-44.0(0.2)	-15.7(0.3)	27.8(0.3)	-27.8(0.1)	51.8(0.1)	-7.9(0.3)
EGCG(2S,3R)	Ring G	-29.0(0.3)	-20.0(0.3)	21.6(0.2)	-21.6(0.1)	39.5(0.1)	-9.5(0.2)
EGCG(2S,3S)	Ring G	-39.3(0.4)	-25.2(0.4)	31.9(0.3)	-26.0(0.1)	50.3(0.1)	-8.3(0.4)

The standard error of the mean of the free energy is shown in parentheses.

**Fig 6:**
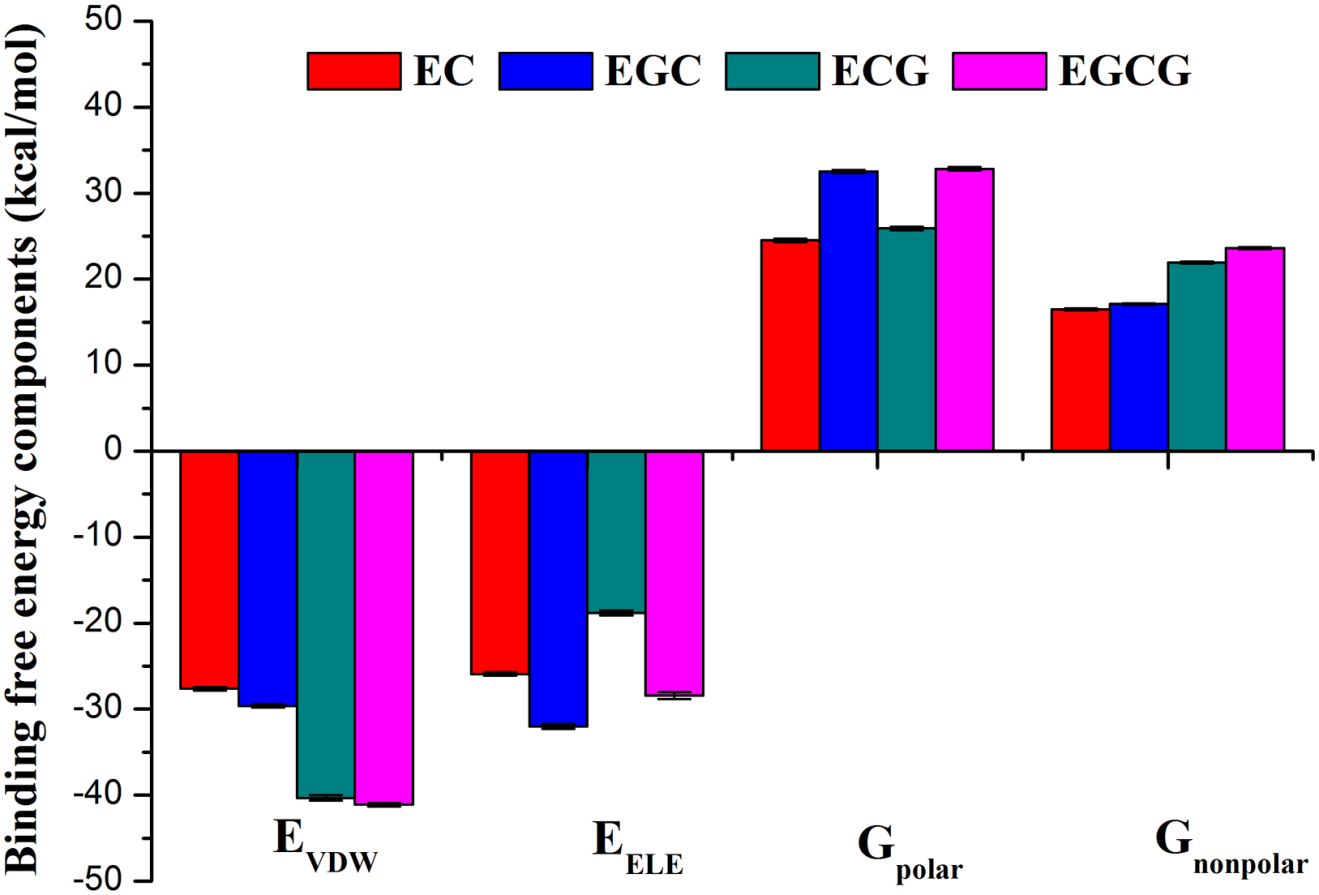
Analysis of contributions of each component in binding free energy. Comparison of the binding free energy components of trypsin binding with EC (red), EGC (blue), ECG (dark cyan) and EGCG (magenta).

In order to shed light on the dominant interaction for driving catechins binding to trypsin, it is essential to decompose the binding free energy into individual energy components. The contributions of each component were presented in Fig 6. The van der Waals interaction (*ΔE*_*vdW*_) and the electrostatic interaction (*ΔE*_*ele*_) in the complex are the favorable for binding, while the polar and the nonpolar solvation terms show unfavorable contributions. The electrostatic interaction and polar solvation free energy are reversely correlated in ECG and EGCG-trypsin complex. It is reasonable considered that the polar solvation screens the electrostatic interactions between trypsin and ECG or EGCG[79]. Usually, the van der Waals interactions and the nonpolar solvation energies are closely correlated with the hydrophobic interactions responsible for the burial of hydrophobic groups of catechins. *ΔE*_*vdW*_ +*ΔG*_*nonpolar*_ are -11.1, -12.5, -18.9 and -17.4 kcal/mol for EC, EGC, ECG and EGCG binding to trypsin, respectively. This shows beneficial contributions for binding free energies, indicating that the hydrophobic interaction drives catechins binding to trypsin. The aromatic ring is responsible for the hydrophobic interaction, thus ECG and EGCG with more aromatic ring have stronger binding affinity than EC and EGC.

**Fig 7:**
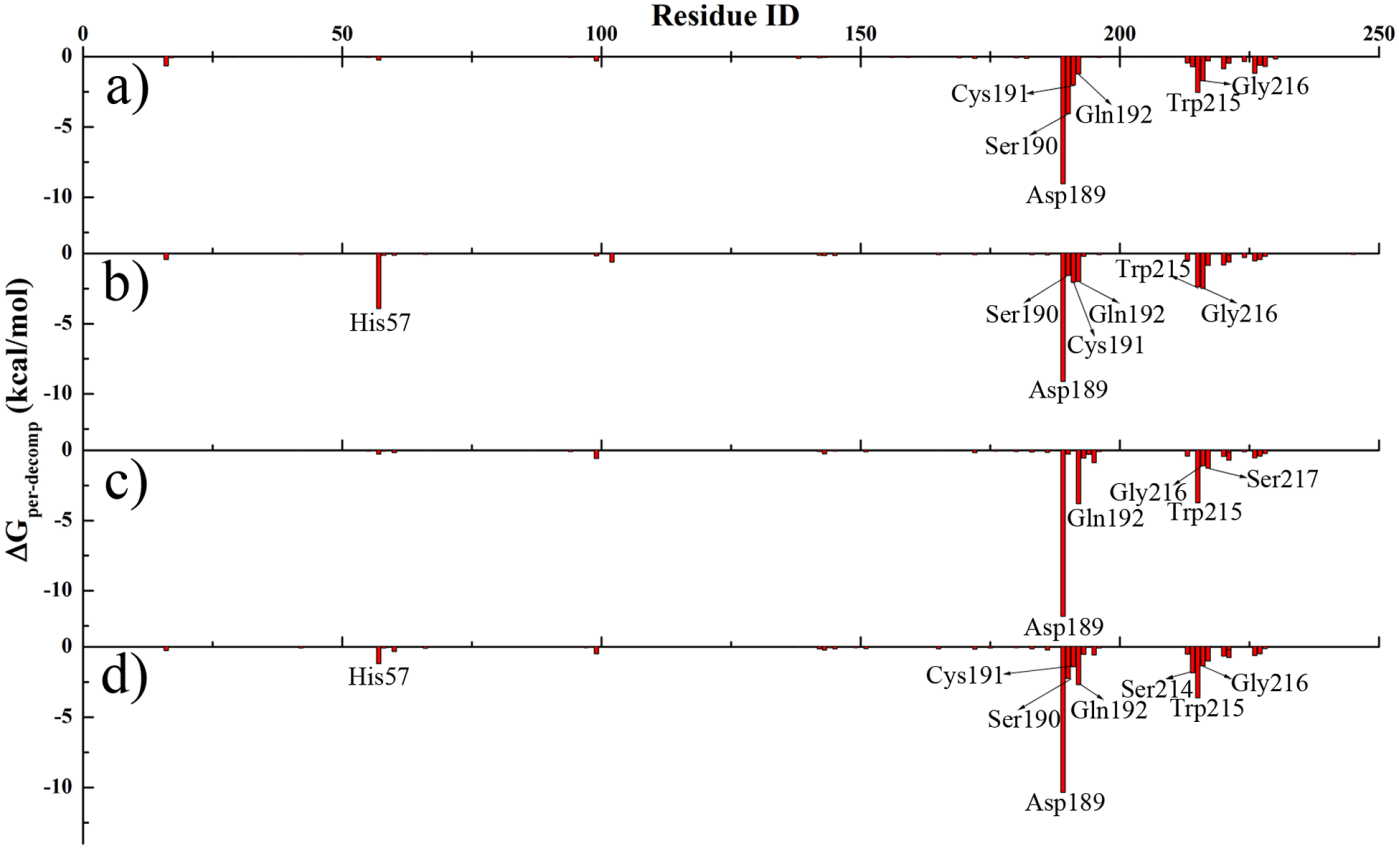
Analysis of contributions of each residue in binding free energy. Binding free energies contributed from each residue to stabilize the trypsin-catechin complex. Residues with *ΔG*_*per-decomp*_ ≥ 1.0 kcal/mol were labeled. (a) trypsin-EC; (b) trypsin-EGC; (c) trypsin-ECG; and (d) trypsin-EGCG.

Further, the contributions of each residue for binding free energy, *ΔG*_*per-decomp*_ was shown in Fig 7. The contributions of van der Waals interaction and the electrostatic interaction ascribe to each residue were also plotted in S6 and S7 Figs. Residues with energies no less than 1.0 kcal/mol were labeled. Residues in or at the vicinity of the three motifs in S1 pocket play an important role in binding catechins to trypsin. Asp189 has strong electrostatic contribution to catechins that overwhelmed the unfavorable van der Waals interaction, and thus becomes the strongest site to bind catechins. Since hydrogen bond is enclosed in electrostatic attraction, further analysis of hydrogen bonding was carried out. A hydrogen bond was defined as the distance of the heavy atoms between donor and acceptor is less than 3.6 Å, and the angle of donor-H-acceptor is no less than 120°. The occurrence and geometry of hydrogen bonds between trypsin and catechin were listed in S2 Table. As illustrated in Fig 4, the side-chain in Asp189 can form two stable hydrogen bonds with catechins, and they are also stable in whole MD simulation with high occurrence. Other residues such as Gln192, Trp215 and Gly216 with strong hydrophobic side-chains exhibit strong van der Waals interaction with catechins. The aromatic ring in Trp215 also provides π-stacking interaction to bind catechins. His57 in the catalytic triad also has strong interaction through hydrogen bond to catechins.

Overall, the binding free energy calculation indicates that hydrophobic interaction together with hydrogen bonding dominates the binding of catechins to trypsin. The van der Waals and electrostatic interaction (majorly from hydrogen bonding) show favorable contributions, while the solvation component has unfavorable contribution in the formation of trypsin-catechin complexes.

## Conclusion

In this work, we investigated the binding of catechins to trypsin using an integration of semi-flexible molecular docking, fully flexible molecular dynamics simulation and free energy calculation. Catechins could bind to the active pocket S1 of trypsin with prone orientations. The binding affinity is dependent on the number and arrangement of hydroxyl and aromatic groups in catechins. The binding free energy is in the order of EGCG > ECG > EC > EGC, and 2R-3R stereoisomer has the strongest binding. Functional groups in catechins are stretched in the binding. Meanwhile, given residue motifs in trypsin, especially those in or at the vicinity of the S1 pocket and the catalytic triad, and the structures of catechins all have synergic conformation change to facilitate the binding. Hydrophobic interaction through the van der Waals interaction, and hydrogen bonding enclosed in electrostatic attraction overwhelmed the unfavorable solvation contribution to stabilize trypsin-catechin complex. These findings could provide a detailed understanding from energetic and structural aspects for protein-ligand binding and a molecular basis for rational design of new potent inhibitors to regulate the bioactivity of trypsin.

## Supporting Information

S1 Fig The steroisomers of EGCG. (TIF)

S2 Fig RMSD of trypsin and EGCG in an 80 ns MD simulation. Time evolutions of the RMSD in an 80 ns MD simulation on the trypsin-EGCG complex for the backbone of trypsin and EGCG.
(TIF)

S3 Fig Docking structures of catechins binding with trypsin. Docking structures of trypsin with a): EC, b): EGC, c): ECG, and d): EGCG. Hydrogen bonds and hydrophobic interactions have important contribution in binding are highlighted. The catalytic triad (Asp102-His57-Ser195) is shown in stick and the ligands are shown in stick-ball.
(TIF)

S4 Fig Docking structure of four steroisomers of EGCG. The docking structure with the superposition of four steroisomers of EGCG in the S1 pocket: 2R, 3R-EGCG (green); 2R, 3S-EGCG (cyan); 2S, 3R-EGCG (magenta); 2S, 3S-EGCG (yellow). Trypsin is represented by cartoon model, while the steroisomers of EGCG are represented by stick model with different size.
(TIF)

S5 Fig RMSD of four types of catechins. Time evolutions of RMSD of four types of catechins with respect to their initially docking positions: EC (red), ECG (blue), EGC (dark cyan) and EGCG (magenta), respectively.
(TIF)

S6 Fig Analysis of contributions of van der Waals interactions. Van der waals interactions (*ΔE*_*vdW*_) contribution spectrum for binding free energy on per-residue basis of trypsin-catechin complex. The residue with |*ΔE*_*vdW*_| ≥ 1.0 kcal/mol is labeled. (a) trypsin-EC; (b) trypsin-EGC; (c) trypsin-ECG; and (d) trypsin-EGCG.
(TIF)

S7 Fig Analysis of contributions of electrostatic interactions. Electrostatic interactions (*ΔE*_*ele*_) contribution spectrum for binding free energy on a per-residue basis of trypsin-catechin complex. The residue with |*ΔE*_*ele*_| ≥ 1.0 kcal/mol is labeled. (a) trypsin-EC; (b) trypsin-EGC; (c) trypsin-ECG; and (d) trypsin-EGCG.
(TIF)

S1 Table The binding affinity and occurrence of four steroisomers of EGCG. This table presents the binding affinity (kcal/mol) and occurrence in the S1 pocket with given orientations for the four stereoisomers of EGCG binding with trypsin based on docking structure models.
(PDF)

S2 Table Analysis of hydrogen bonds between trypsin and catechins. This table presents the occurrence and the geometry of hydrogen bonds between trypsin and catechin based on MD simulation trajectories. The occurrence was counted against 2000 structure models.
(PDF)
