# Supplementary figures and images for: Investigate the Binding of Catechins to Trypsin Using Docking and Molecular Dynamics Simulation
Source: PLoS One. 2015 May 4;10(5):e0125848. doi: 10.1371/journal.pone.0125848 (PMC4418572; doi:10.1371/journal.pone.0125848)

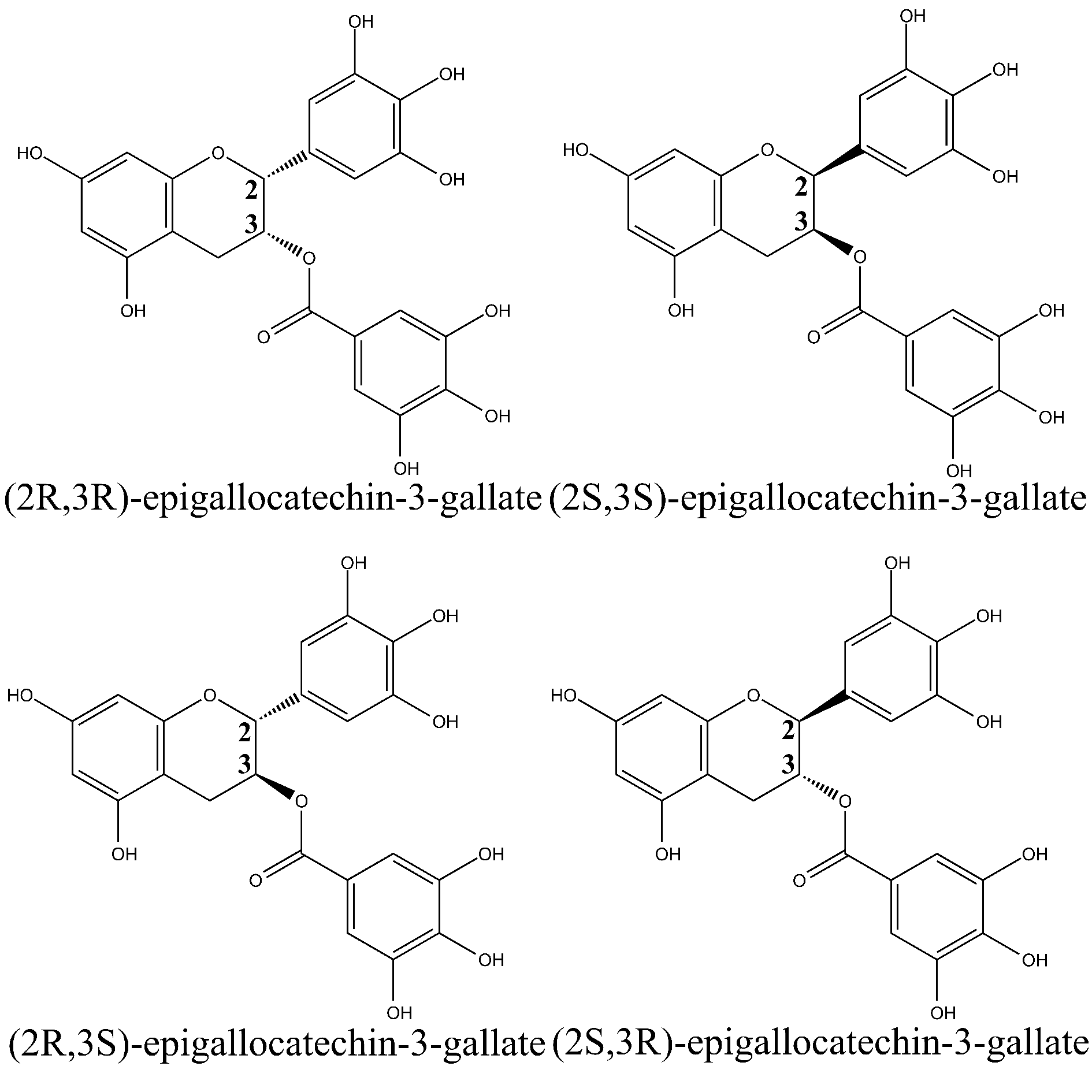

Supplement: S1 Fig — (TIF) [file pone.0125848.s001.tif]

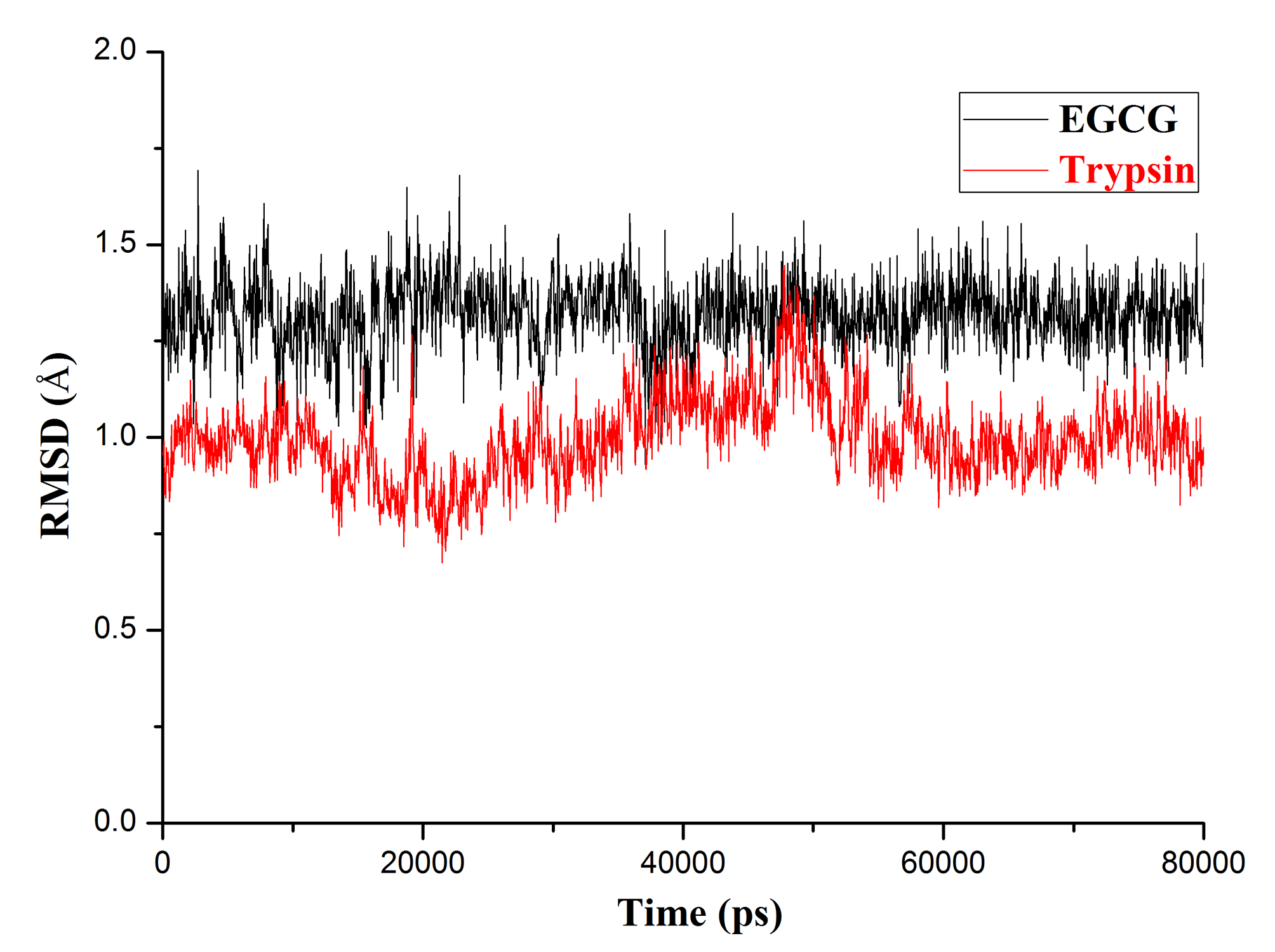

Supplement: S2 Fig — Time evolutions of the RMSD in an 80 ns MD simulation on the trypsin-EGCG complex for the backbone of trypsin and EGCG. (TIF) [file pone.0125848.s002.tif]

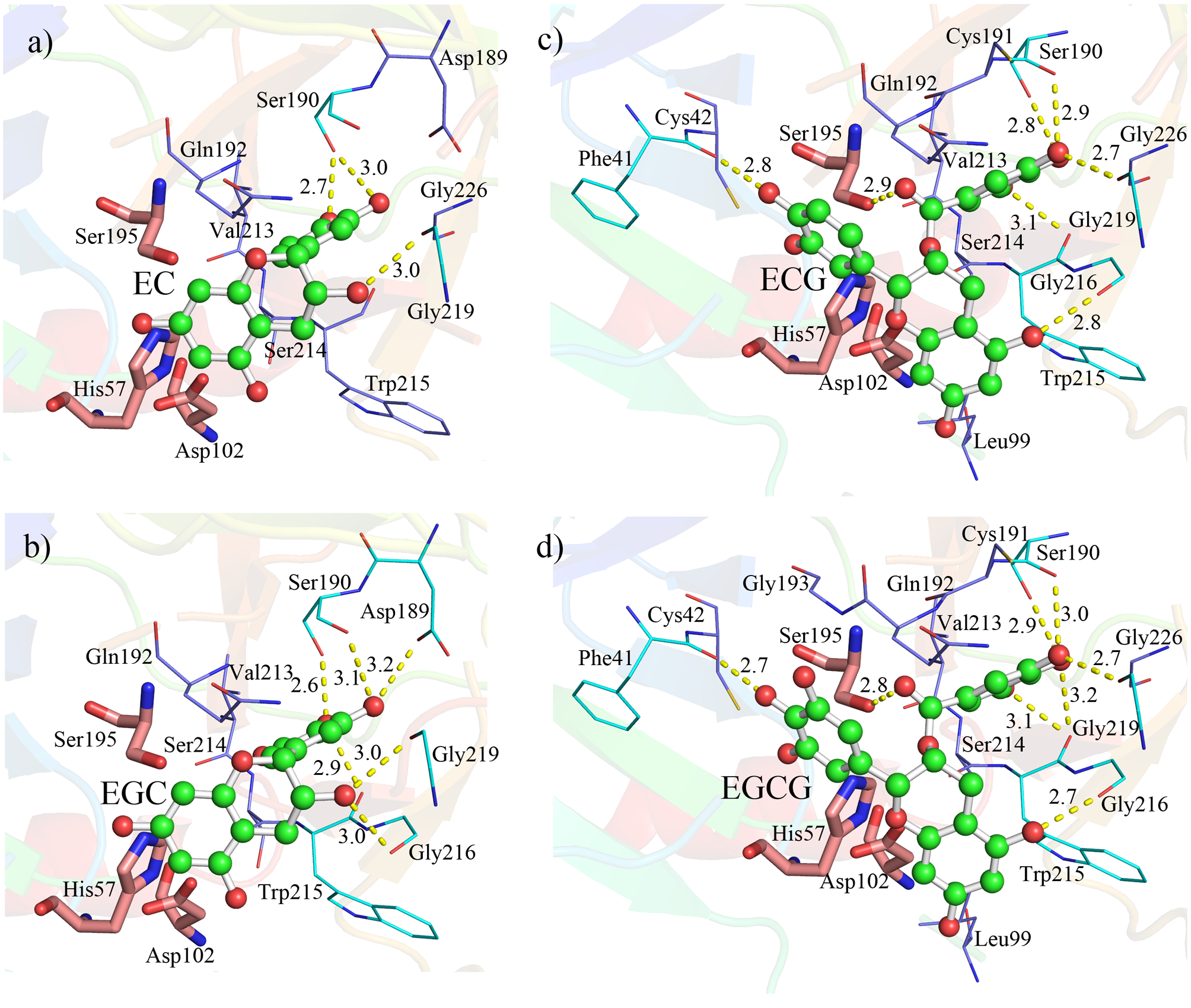

Supplement: S3 Fig — Docking structures of trypsin with a): EC, b): EGC, c): ECG, and d): EGCG. Hydrogen bonds and hydrophobic interactions have important contribution in binding are highlighted. The catalytic triad (Asp102-His57-Ser195) is shown in stick and the ligands are shown in stick-ball. (TIF) [file pone.0125848.s003.tif]

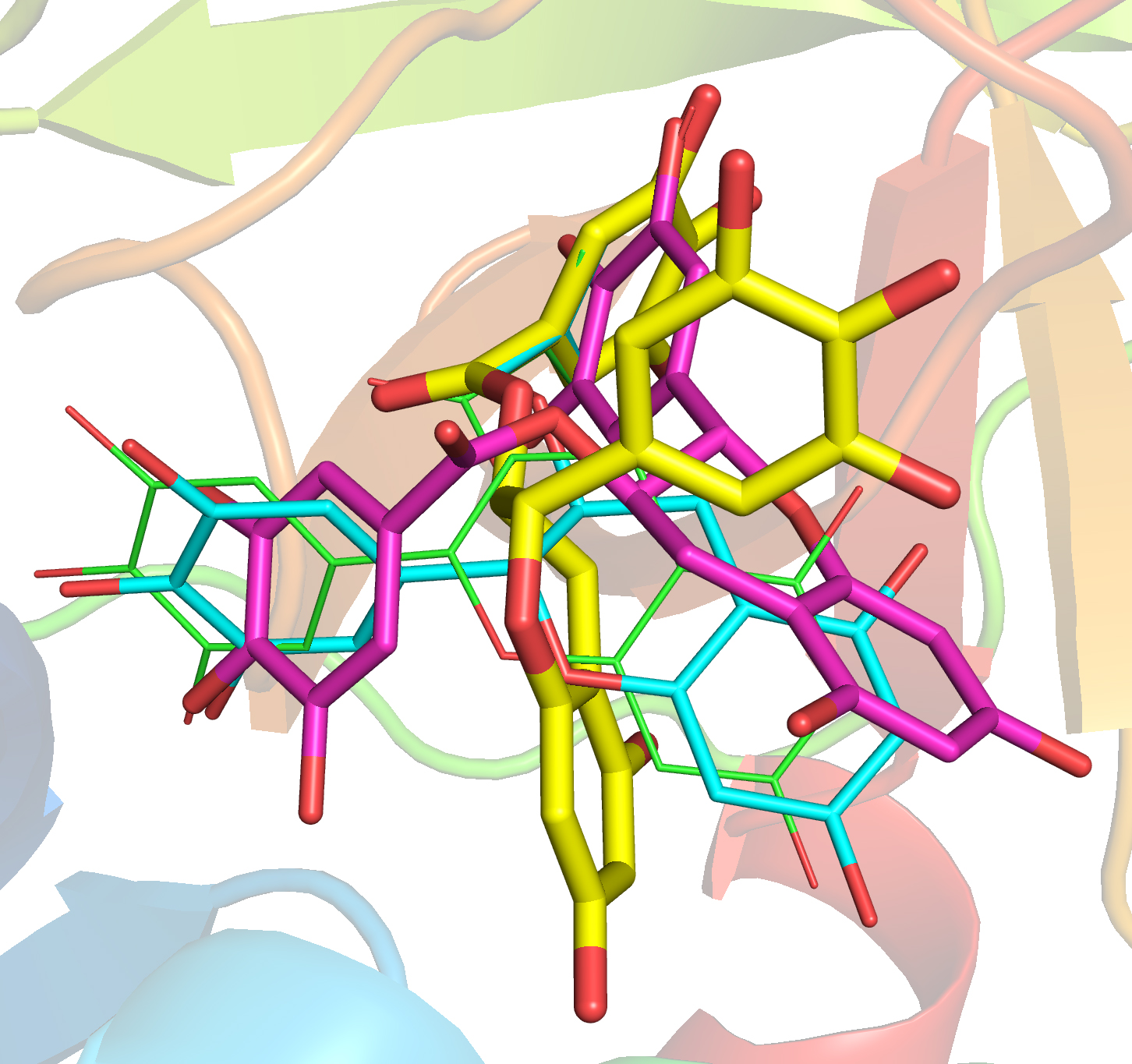

Supplement: S4 Fig — The docking structure with the superposition of four steroisomers of EGCG in the S1 pocket: 2R, 3R-EGCG (green); 2R, 3S-EGCG (cyan); 2S, 3R-EGCG (magenta); 2S, 3S-EGCG (yellow). Trypsin is represented by cartoon model, while the steroisomers of EGCG are represented by stick model with different size. (TIF) [file pone.0125848.s004.tif]

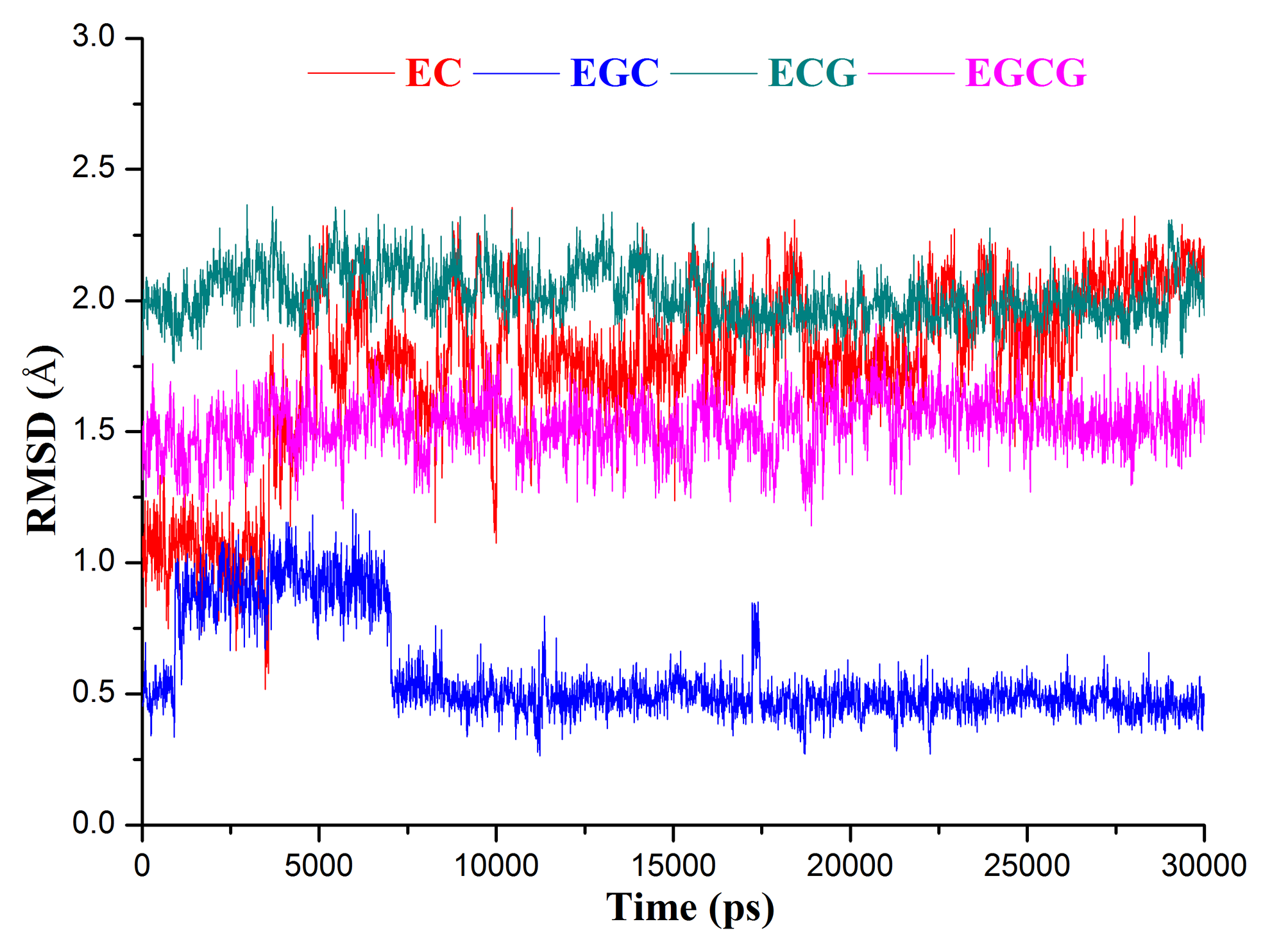

Supplement: S5 Fig — Time evolutions of RMSD of four types of catechins with respect to their initially docking positions: EC (red), ECG (blue), EGC (dark cyan) and EGCG (magenta), respectively. (TIF) [file pone.0125848.s005.tif]

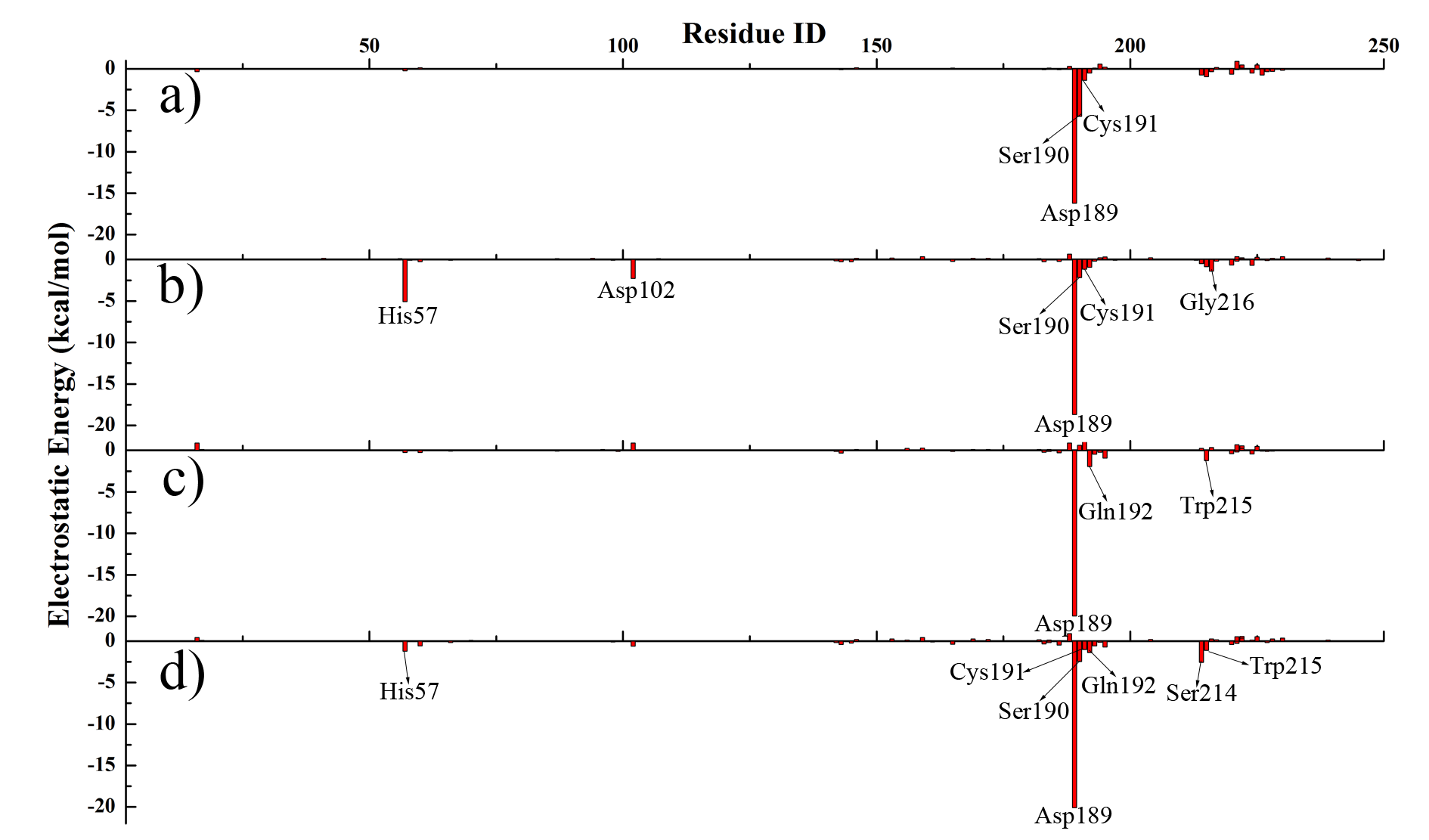

Supplement: S6 Fig — Van der waals interactions (ΔE vdW) contribution spectrum for binding free energy on per-residue basis of trypsin-catechin complex. The residue with |ΔE vdW| ≥ 1.0 kcal/mol is labeled. (a) trypsin-EC; (b) trypsin-EGC; (c) trypsin-ECG; and (d) trypsin-EGCG. (TIF) [file pone.0125848.s006.tif]

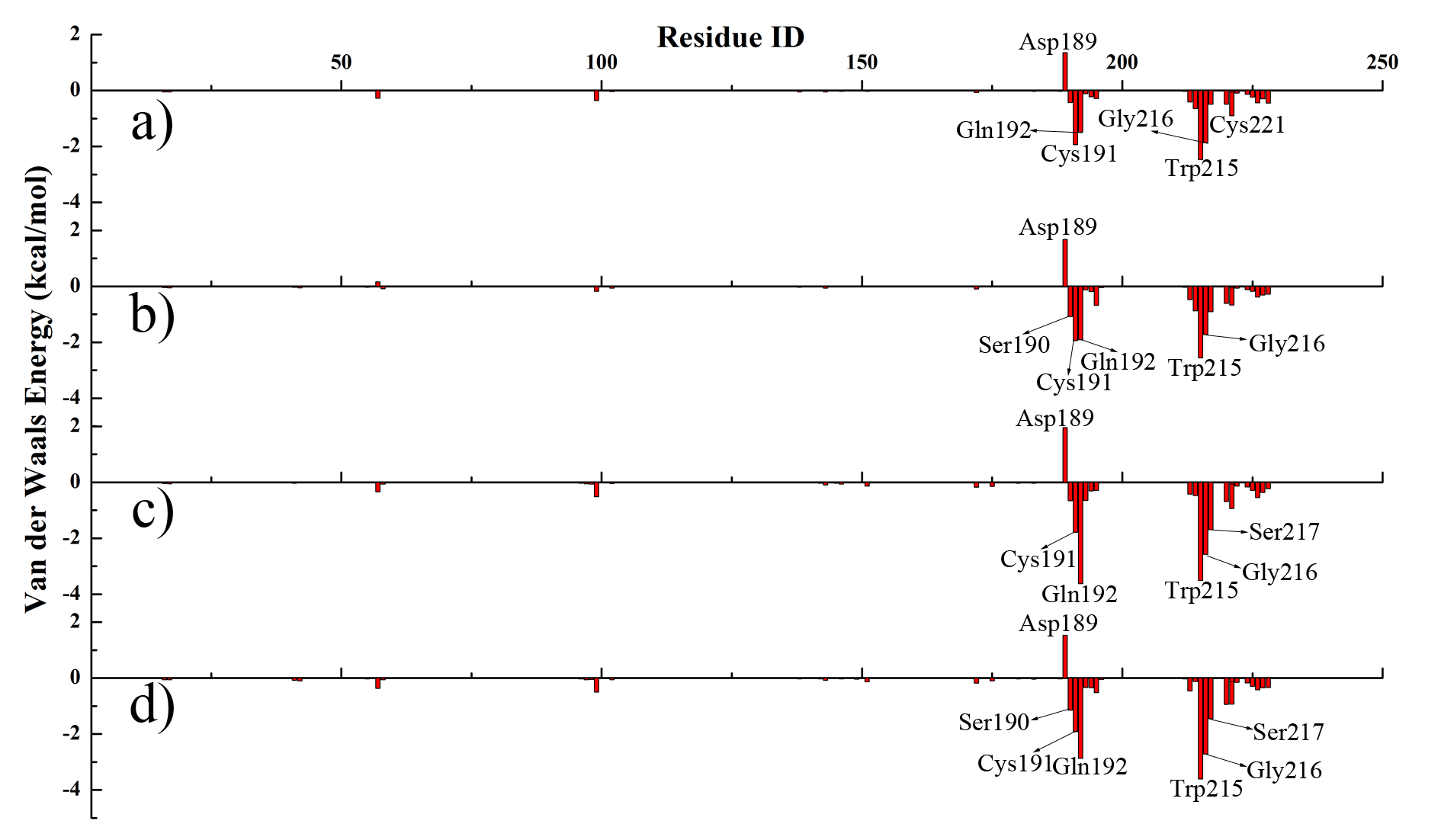

Supplement: S7 Fig — Electrostatic interactions (ΔE ele) contribution spectrum for binding free energy on a per-residue basis of trypsin-catechin complex. The residue with |ΔE ele| ≥ 1.0 kcal/mol is labeled. (a) trypsin-EC; (b) trypsin-EGC; (c) trypsin-ECG; and (d) trypsin-EGCG. (TIF) [file pone.0125848.s007.tif]
