# Supplementary material for: Investigate the Binding of Catechins to Trypsin Using Docking and Molecular Dynamics Simulation
Source: PLoS One. 2015 May 4;10(5):e0125848. doi: 10.1371/journal.pone.0125848 (PMC4418572; doi:10.1371/journal.pone.0125848)
Supplement: S1 Table — This table presents the binding affinity (kcal/mol) and occurrence in the S1 pocket with given orientations for the four stereoisomers of EGCG binding with trypsin based on docking structure models. (PDF) [file pone.0125848.s008.pdf]

Table S1. The binding affinity and occurrence of four steroisomers of EGCG. This table presents the binding affinity (kcal/mol) and occurrence in the S1 pocket with given orientations for the four stereoisomers of EGCG binding with trypsin based on docking structure models.

	2R,3R-EGCG (100.0%[a])	2R,3S-EGCG (85.0%[a])	2S,3R-EGCG (75.0%[a])	2S,3S-EGCG (100.0%[a])
Ring G	-8.5(45.0%[b])	-8.3(41.2%[b])	-7.7(46.7%[b])	-8.1(30.0%[b])
Ring B	-8.3(40.0%[b])	-7.7(47.1%[b])	-7.8(40.0%[b])	-7.5(45.0%[b])
Ring AC	-7.5(15.0%[b])	-7.4(11.7%[b])	-7.8(13.3%[b])	-7.6(25.0%[b])

[a] The number of conformations of four types of catechins binding to the S1 pocket in all top 20 docking pose.

[b] The number of conformations of different group preferentially enter into the S1 pocket in those poses that catechins were bound to the S1 pocket.
