# Supplementary material for: Investigate the Binding of Catechins to Trypsin Using Docking and Molecular Dynamics Simulation
Source: PLoS One. 2015 May 4;10(5):e0125848. doi: 10.1371/journal.pone.0125848 (PMC4418572; doi:10.1371/journal.pone.0125848)
Supplement: S2 Table — This table presents the occurrence and the geometry of hydrogen bonds between trypsin and catechin based on MD simulation trajectories. The occurrence was counted against 2000 structure models. (PDF) [file pone.0125848.s009.pdf]

Table S2. The analysis of hydrogen bonds between trypsin and catechins.

This table presents the occurrence and the geometry of hydrogen bonds between trypsin and catechin based on MD simulation trajectories. The occurrence was counted against 2000 structure models.

System	Donor	Acceptor	Occurrence(%)	Distance (Å)	Angle (°)
EC	Ring B OH	Asp189 OD1	99.8	2.642 (0.11)	14.97 (9.05)
	Ring B OH	Asp189 OD1	99.6	2.758 (0.17)	15.34 (9.03)
	Ring B OH	Asp189 OD2	53.8	3.158 (0.22)	52.98 (5.87)
EGC	Ring A OH	His57 NE2	97.2	2.796 (0.12)	15.50 (8.62)
	Ring B OH	Asp189 OD1	79.4	2.972 (0.31)	30.74 (18.89)
	Ring B OH	Asp189 OD2	74.55	2.877 (0.32)	25.09 (17.81)
	Ring C OH	Gly216 O	66.3	2.817 (0.18)	26.18 (13.71)
	Ring B OH	Asp189 OD2	52.8	2.600 (0.12)	15.33 (9.92)
	Ring B OH	Asp189 OD1	42.4	2.615 (0.16)	16.85 (11.38)
ECG	Ring G OH	Asp189 OD1	99.8	2.670 (0.12)	19.95 (11.08)
	Ring G OH	Asp189 OD1	94.9	2.851 (0.28)	26.15 (16.60)
	Ring G OH	Asp189 OD2	83.1	2.883 (0.35)	36.40 (15.96)
	Ring B OH	Ser195 OG	33.4	2.894 (0.21)	22.70 (13.72)
EGCG	Ring G OH	Asp189 OD1	90.7	2.813 (0.25)	19.14 (14.57)
	Ring G OH	Asp189 OD1	81.7	2.622 (0.13)	16.25 (10.69)
	Ring G OH	Asp189 OD2	58.6	3.124 (0.32)	39.13 (17.51)
	Ring B OH	Ser214 O	42.5	2.662 (0.12)	18.79 (9.46)
	Ring B OH	His57 NE2	40.1	2.977 (0.20)	29.54 (10.69)
	Ring B OH	Ser195 OG	31.5	2.863 (0.19)	26.63 (12.21)
